# Copeptin is associated with mortality and outcome in patients with acute intracerebral hemorrhage

**DOI:** 10.1186/1471-2377-10-34

**Published:** 2010-05-26

**Authors:** Christian Zweifel, Mira Katan, Philipp Schuetz, Martin Siegemund, Nils G Morgenthaler, Adrian Merlo, Beat Mueller, Mirjam Christ-Crain

**Affiliations:** 1 Department of Neurosurgery, University Hospital of Basel, Spitalstrasse 21, 4031 Basel, Switzerland; 2 Department of Neurology, University Hospital of Basel, Petersgraben 4, 4031 Basel, Switzerland; 3 Department of Endocrinology and Clinical Nutrition, University Hospital of Basel, Petersgraben 4, 4031 Basel, Switzerland; 4 Department of Anesthesia, Operative Intensive Care Unit, University Hospital Basel, Spitalstrasse 21, 4031 Basel, Switzerland; 5 Department of Research, BRAHMS Aktiengesellschaft, Biotechnology Centre, Neuendorfstrasse 25,16761 Hennigsdorf, Germany; 6 Department of Internal Medicine, Haus 7, Tellstrasse, 5001 Aarau, Switzerland

## Abstract

**Background:**

Spontaneous intracerebral hemorrhage (ICH) accounts for a high mortality and morbidity. Early prediction of outcome is crucial for optimized care and treatment decision. Copeptin, the C-terminal part of provasopressin, has emerged as a new prognostic marker in a variety of diseases, but its prognostic value in ICH is unknown.

**Methods:**

In 40 consecutive patients who were admitted to the hospital within 72 hours after a spontaneous ICH, the plasma copeptin level was measured with a sandwich immunoassay upon admission. The prognostic value of copeptin to predict 30 day mortality and functional outcome after 90 days was assessed. A favorable outcome was defined as a Barthel score above 85 and a score below 3 on the Modified Rankin Scale.

**Results:**

Copeptin correlated positively with hematoma volume (r = 0.32, p < 0.05) and negatively with the Glasgow Coma Scale (GCS) on admission (r = -0.35, p < 0.05). Copeptin levels were higher in patients who died within 30 days than in 30-day survivors (179.0 pmol/l (IQR 33.7- 566.0) vs. 12.9 pmol/l (IQR 5.2 - 42.8), p = 0.003). Copeptin levels were also higher in patients with an unfavorable functional outcome at 90 days compared to patients with a favorable outcome (32.4 pmol/l (IQR 9.5-97.8) vs. 11.9 pmol/l (IQR 3.2-19.8), p = 0.04). For the prediction of death, receiver-operating-characteristics analysis revealed an area under the curve (AUC) for copeptin of 0.88 (95%CI 0.75-1.00). The predictive value of the copeptin concentration was thus similar to that of GCS (AUC 0.82 (95%CI 0.59-1.00) p = 0.53), of the ICH Score (AUC 0.89, (95%CI 0.76-1.00), p = 0.94) and the ICH Grading Scale (AUC 0.86 (95%CI 0.69-1.00), p = 0.81).

**Conclusions:**

Copeptin is a new prognostic marker in patients with an ICH. If this finding can be confirmed in larger studies, copeptin might be an additional valuable tool for risk stratification and decision-making in the acute phase of ICH.

**Trial Registration:**

(**Clinical Trial Registration**: ISCTRN00390962)

## Background

Intracerebral hemorrhage (ICH) is more fatal and disabling than ischemic stroke and ranges from 10 to 20 cases per 100,000 population reflecting 10 to 15 percent of all stroke patients [1]. Early prognostication of the risk of death or of a poor long-term outcome would enable optimized care and improved allocation of health-care resources. Several scales of outcome prediction after primary ICH have been proposed [2]. Dynamic factors like hematoma volume expansion, edema formation or persistent high blood pressure are known to be associated with early neurologic deterioration and poor outcome[3]. In combination with the clinical findings, a readily measurable predictive marker predicting mortality in patients with ICH would be helpful for early prognostication and risk stratification. Biomarkers are attracting increasing attention as potential predictors of outcome in ischemic and hemorrhagic stroke [4,5].

Copeptin, the C-terminal portion of provasopressin, is a 39-amino acid glycopeptide that has been found to be a stable and sensitive surrogate marker for vasopressin (AVP) release [6]. As AVP is a potent synergistic factor of the hypothalamo-pituitary-adrenal axis, copeptin might act as a marker of the individual stress response. In fact, it has been shown that copeptin measurement is useful for prognostic assessment in patients with cardiovascular diseases, lower respiratory tract infection, sepsis and head injury [7,8]. Copeptin levels have also been found to be elevated in ischemic stroke patients; in this group of patients, high copeptin levels were highly predictive for poor functional outcome and mortality[9].

We tested the hypothesis that high copeptin levels in acute hemorrhagic stroke patients are also associated with mortality and poor functional outcome.

## Methods

### Study design and setting

This is a prospective study evaluating copeptin concentrations in consecutive patients with hemorrhagic stroke admitted to the Emergency Department of the University Hospital of Basel, Switzerland from November 2006 until November 2007 [9]. The study was approved by the local ethical committee for human studies (EKBB) and registered in the ISCTRN database (ISCTRN 00390962 and ClincalTrials.gov number NCT00390962). Informed consent was obtained from the patients or their next of kin before enrolment.

### Participants

Forty consecutive patients who were admitted to the emergency department with spontaneous ICH within 72 hours of symptom onset were prospectively analyzed. Patients with a subarachnoid hemorrhage or traumatic ICH were not included.

### Neuroimaging

On the initial CT scan, the ICH volume was assessed with the *ABC/2 *method[10]. In this method, *A *is the greatest diameter on the largest hemorrhage slice, *B *is the diameter perpendicular to *A*, and *C *is the number of axial slices with hemorrhage multiplied by the slice thickness.

### Clinical variables and follow up

Clinical status and severity of disease were assessed on admission. For clinical assessment, the Glasgow Coma Scale (GCS) was used, and relevant co-morbidities were assessed with the Charlson co-morbidity index [11,12]. For the assessment of 30-day mortality and functional outcome at 90 days, structured interviews were carried out by a trained medical student who was blinded to copeptin levels. Functional outcomes were measured with the Barthel Index (BI) [13] and Modified Rankin Scale (mRS)[14]. A favorable outcome was defined as a BI score above 85 and a score below 3 on the mRS. To compare the predictive value of copeptin with combined clinical features, the ICH Score according to Hemphill [15] and the ICH Grading Scale according to Ruiz-Sandoval [16] was used.

### Endpoints

The primary endpoint of this study was the predictive value of copeptin for 30-day mortality in patients with hemorrhagic stroke. 30-day mortality is a common endpoint in prognostic ICH studies[2]. The secondary endpoint was the functional outcome at 90 days, as measured by the BI and the mRS.

### Assays

Results of the routine blood analyses including sodium concentrations, blood glucose, white blood cells, C-reactive protein (CRP) (mg/ml) and serum osmolarity were consecutively recorded in all patients. Immediately on admission plasma was collected in plastic tubes containing ethylenediaminetatraacetic acid (EDTA). They were placed on ice and then centrifuged at 3000 g and plasma was frozen at -70°C until batch-analysis. Copeptin levels were measured with a chemiluminescens sandwich immunoassay with a lower detection limit of the assay of 0.4 pmol/l[17]. In healthy volunteers and under rest, normal copeptin levels have 4.0-4.4 pmol/l as range with only 5% of values lying outside this range [6].

### Statistical analysis

A logarithmic transformation was performed to obtain a normal distribution for skewed variables (i.e. copeptin concentrations). Discrete variables were expressed as counts (percentage) and continuous variables as medians and interquartile ranges (IQR) unless stated otherwise. Frequency comparisons were performed with the chi-square test. Two-group comparisons were performed with the Mann-Whitney-U test if only two groups were compared and the Kruskal-Wallis one-way analysis of variance was used if more than two groups were being compared. Univariate regression models were calculated to compare the prognostic accuracy of copeptin levels with that of other prognostic parameters. Because of the small number of outcomes, it was not reasonable to perform a multivariate analysis. Receiver-operating-characteristics (ROC) were calculated with the area under the curve (AUC) as an overall prognostic measure. All non-linear data were log transformed before being entered into the logistic regression models. To estimate the potential clinical relevance of copeptin for the prediction of mortality, we calculated Kaplan-Meier survival curves and stratified patients on the basis of the median copeptin level, then used the log-rank test to compare the two groups. Correlation analyses were performed with Spearman rank correlation. All testing was two-tailed, and P values less than 0.05 were considered to indicate statistical significance. All calculations were performed with STATA 9.2 (Stata Corp, College Station, Texas).

## Results

### Baseline Data

The median age of the 40 patients (45% female) was 71 years (IQR 64-78 years), the median GCS on admission was 14 (IQR 13-15). The distribution of the hemorrhage sites were as follows: lobar (47.5%), basal ganglia (45%) and infratentorial (7.5%). The median hematoma volume was 17.8 ml (IQR 6.3-36.3). Median copeptin values of our cohort were 16.3 (IQR 6.3-54.3) pmol/l. The time from symptom onset to blood withdrawal for copeptin determination ranged from 2 to 72 hours. For 7 patients, blood withdrawal was done in the first three hours, for 6 patients between 3 and 6 hours, for 7 between 6 and 12 hours, for 9 patients between 12 and 24 hours and for 11 patients between 24 and 72 hours. Copeptin levels were not significantly different between these groups. Copeptin correlated furthermore with ICH volume (r = 0.32, p < 0.05), with GCS (r = -0.35, p < 0.03) and blood glucose (r = 0.53, p = 0.0008) on admission.

Four patients underwent hematoma evacuation, 3 patients received a ventricular drainage. In most cases, ICH was presumed to be due to uncontrolled hypertension. 9 patients (22.5%) had been taking antiplatelets or anticoagulant drugs before the ICH occurred.

### Primary Endpoint

**Figure 1:**
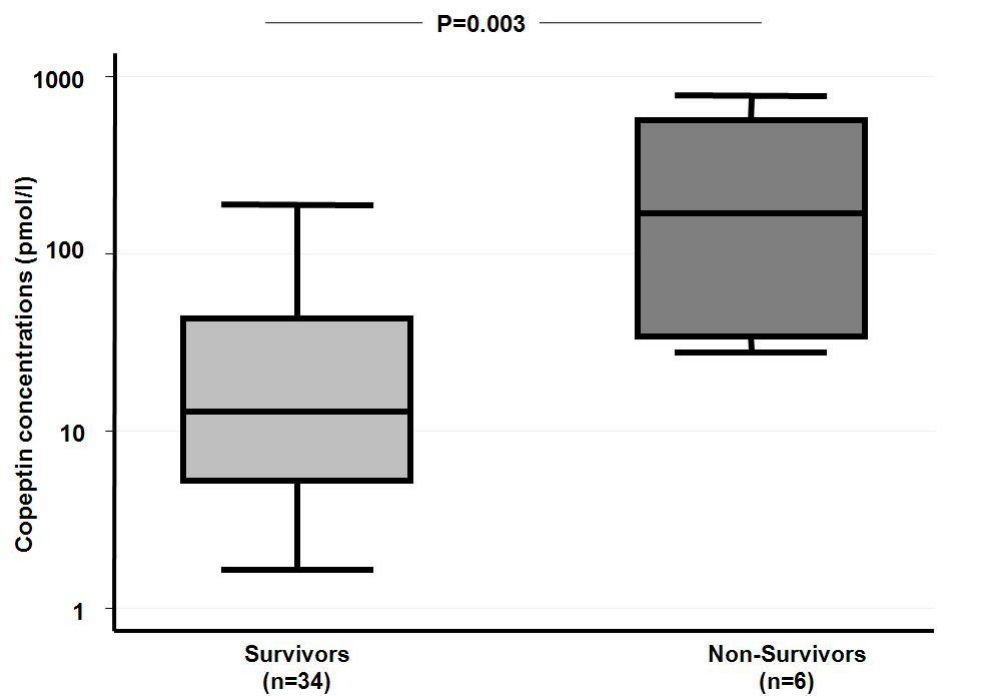
**Baseline log copeptin levels in patients who died or survived after ICH**. A box encloses the middle 50 percent, where the median is represented as a horizontal line inside the box.

On follow-up at 30 days, 6 patients had died, yielding a 30-day mortality of 15%. None of the patients had died due to withdrawal of care. Table 1 shows the baseline characteristics of the survivors and non-survivors on admission. The non-survivors tended to be older, and were more frequently female. Neurological examination on admission revealed a lower GCS level in non-survivors than in survivors (10 pmol/l (IQR 5-13) vs. 14 pmol/l (IQR 14-15), p = 0.009). Copeptin levels were significantly higher in non-survivors than in survivors (179.0 pmol/l (IQR 33.7- 566.0) vs. 12.9 pmol/l (IQR 5.2-42.8), p = 0.003) (Figure 1). In univariate logistic regression analysis, only GCS, the volume of the ICH, and the copeptin level were significant predictors of mortality (Table 2). The overall prognostic accuracy of copeptin as assessed in ROC curve analysis (AUC 0.88 (95%CI 0.75-1.00) was comparable to that of the GCS (AUC 0.82 (95%CI 0.59-1.00), p = 0.56), of the ICH Score[15] (AUC 0.89, (95%CI 0.76-1.00), p = 0.94) and the ICH Grading Scale[16] (AUC 0.86 (95%CI 0.69-1.00), p = 0.81).

**Table 1:** Baseline characteristics of ICH Cohort (n = 40)

	Survivors	Non-Survivors	*p*
	(n = 34)	(n = 6)	
**Demographics**			
Age (years)	69.5 (61.5 - 75.8)	80 (73.3 - 82.3)	*0.06*
Gender (Female)	38% (13)	80% (5)	***0.04***
			
**Clinical parameters**			
GCS*	15 (14-15)	10 (4-13)	***0.009***
Charlson Index	1 (0-1)	1.5 (1-2)	*0.18*
Body Temperature (C°)	37.1 (36.7-37.4)	36.0 (35.9-37.5)	*0.32*
Hematoma Volume in ml	13 (5-30)	69 (60-75)	***0.003***
Hypertension	67.7% (23)	100% (6)	*0.444*
Drugs (antiplatelets, anticoagulants)	26.5% (9)	0	*NA*
ICH Score	1 (0-1)	3 (2.5-4)	***0.002***
ICH Grading Scale	7 (7-8)	9.5 (9-11)	***0.004***
			
Location			
Basal ganglia	44.1% (15)	50% (3)	*0.75*
Lobar	47.1% (16)	50% (3)	*0.72*
Infratentorial	8.8% (3)	0	*NA*§
			
Presence of IVH†	20.6% (7)	33.3% (2)	*0.602*
			
Operation			
Surgical hematoma evacuation	8.8% (3)	16.7% (1)	*0.656*
EVD‡ Placement	8.8% (3)	0	*NA*
			
**Laboratory Values**			
Sodium (mmol/l)	138 (136-141)	139 (132-141)	*0.89*
Osmolarity (mosml/l)	297 (293-301)	292 (283-300)	*0.53*
Glucose (mmol/l)	6.6 (5.8-7.8)	7.7 (6.1-8.4)	*0.64*
White blood count (×10^9^/l)	9.5 (6.7-11.6)	8.7 (7.5-8.8)	*0.58*
C-reactive Protein (mg/l)	6 (3-18)	12 (3-81)	*0.54*
Copeptin level (nmol/l)	12.9 (5.2- 42.8)	179.0 (33.7-566.0)	***0.003***

Values are presented as median (lower quartile, upper quartile) or % (counts)

* GCS = Glasgow Coma Scale; † IVH = intraventricular hemorrhage, ‡ EVD = external ventricular drainage; § NA = not available; ICH Score according to Hemphill [15]; ICH Grading Scale according to Ruiz-Sandoval [16]

**Table 2:** Prediction of 30 Day mortality (n = 6) in univariate analysis of all patients (n = 40)

Parameter*	Odds Ratio	95% CI		*p*
Age	1.11	1.00	1.23	*0.054*
Gender	8.08	0.85	77.07	*0.07*
GCS	0.74	0.58	0.93	***0.01***
Charlson Index	1.11	0.71	1.17	*0.65*
Body Temperature	0.58	0.17	2.01	*0.39*
Hematoma Volume	1.05	1.01	1.08	***0.006***
ICH Score	3.92	1.49	10.23	***0.005***
ICH Grading Scale	2.94	1.34	6.42	***0.007***
Sodium	0.94	0.69	1.29	*0.709*
Osmolarity	0.87	0.69	1.10	*0.256*
Glucose	1.15	0.66	1.99	*0.62*
CRP	1.03	0.98	1.07	*0.221*
WBC	0.87	0.63	1.19	*0.38*
Copeptin	19.48	2.10	180.64	***0.009***

* Note that the odds ratio corresponds to a unit increase in the explanatory variable; for copeptin this corresponds to an increase per unit of the log-transformation of copeptin (thus a log transformed increase of 1 corresponds to a copeptin increase of 10 pmol/l). ICH Score according to Hemphill [15]; ICH Grading Scale according to Ruiz-Sandoval [16]

**Figure 2:**
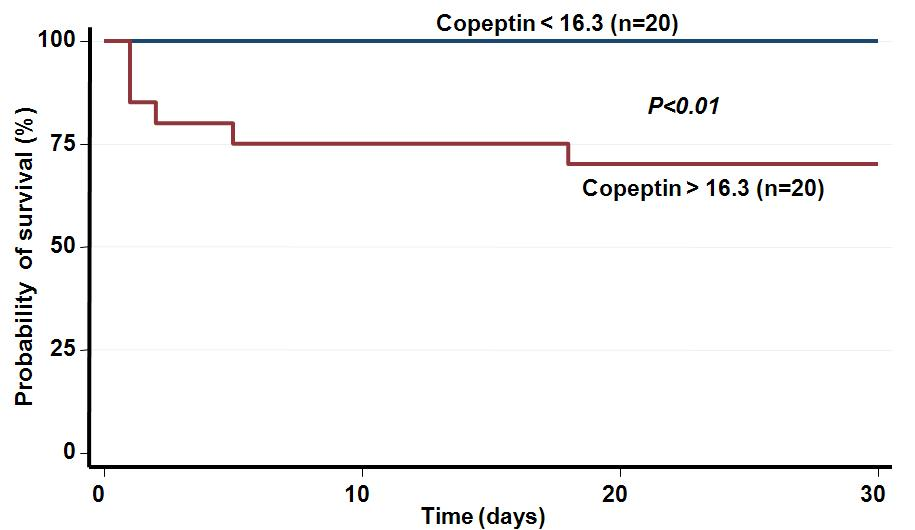
**Kaplan Meyer survival curves for copeptin**.

To illustrate the prognostic value of copeptin in predicting mortality, we calculated Kaplan-Meier survival curves and divided the patients into two groups depending on whether their copeptin level was above or below the median value (16.3 pmol/l). As shown in Figure 2, patients with copeptin levels above the median were significantly more likely to die within 30 days (p < 0.01). These patients had also on admission a significantly lower GCS (13.5 vs 15, p = 0.02), higher plasma glucose (7.6 vs 5.9 mmol/l, p = 0.03) and higher white blood count (WBC, 9.1 vs 8.5 × 10^9^/l, p = 0.01). All other baseline parameters were not significantly different.

### Functional outcome

On day 90, 18 patients (45%) had a favorable outcome, which was defined as Barthel score of 85 or more and a Modified Rankin score below 3, while 22 patients (55%) had an unfavorable outcome. Copeptin levels on admission were significantly higher in patients with an unfavorable outcome (32.4 (IQR 9.5-97.8) vs. 11.9 (IQR 3.2-19.8), p = 0.04). Age, GCS, the volume of ICH, and copeptin levels were significant predictors of functional outcome in univariate logistic regression analysis (Table 3). In ROC analysis, copeptin had an AUC of 0.68 (95%CI 0.52-0.86) which did not differ to any statistically significant extent from the AUC of lesion size (AUC 0.75 (95%CI 0.60-0.91), p = 0.28) or of GCS (AUC 0.81 (95%CI 0.68-0.94), p = 0.24), the ICH Score [15] (AUC 0.83, (95%CI 0.71-0.95), p = 0.14) and the ICH Grading Scale[16] (AUC 0.81 (95%CI 0.68-0.93), p = 0.21).

**Table 3:** Prediction of adverse 90 days outcome defined as a Barthel score < 85 points and mRS < 3 (n = 22) in univariate analysis of all patients (n = 40)

Parameter*	Odds Ratio	95% CI		*p*
Age	1.09	1.01	1.16	***0.023***
Gender	2.40	0.66	8.72	*0.184*
GCS	0.42	0.19	0.94	***0.036***
Charlson Index	1.88	0.96	3.68	*0.07*
Body Temperature	0.54	0.21	1.39	*0.20*
Hematoma Volume	1.06	1.01	1.10	***0.017***
ICH Score	5.48	1.81	16.48	***0.002***
ICH Grading Scale	2.84	1.34	5.98	***0.006***
Sodium	1.29	0.99	1.68	*0.062*
Osmolarity	1.15	0.97	1.35	*0.098*
Glucose	0.99	0.66	1.51	*0.98*
CRP	1.04	0.98	1.10	*0.188*
WBC	1.03	0.86	1.24	*0.73*
Copeptin	3.10	1.02	9.39	***0.046***

* Note that the odds ratio corresponds to a unit increase in the explanatory variable; for copeptin this corresponds to an increase per unit of the log-transformation of copeptin (thus a log transformed increase of 1 corresponds to a copeptin increase of 10 pmol/l). ICH Score according to Hemphill [15]; ICH Grading Scale according to Ruiz-Sandoval [15].

## Discussion

In this prospective study, we demonstrate for the first time that serum copeptin levels measured on admission are associated with 30-day mortality and 90-day functional outcome after ICH.

Copeptin is co-synthesized with vasopressin in the hypothalamus and is released into the portal circulation of the neurohypophysis. Vasopressin contributes to the regulation of osmotic and cardiovascular homeostasis[18,19]. In addition, vasopressin activates the hypothalamo-pituitary-adrenal axis through potentiation of corticotropin-releasing-hormone-induced ACTH secretion and thus reflects the individual stress response at a hypothalamic level[20-22]. Copeptin is known to have prognostic value in a variety of diseases, as it reflects disease severity and thus the chance of recovery. For example, copeptin levels are independent predictors of survival in critically ill patients suffering from hemorrhagic and septic shock [23]. Furthermore, copeptin levels have prognostic implications in patients with acute myocardial infarction and in patients with acute heart failure[24,25]. Therefore, it has been hypothesized that the close and reproducible relation of copeptin levels to the degree of activation of the stress axis is the basis of its unique usefulness as a prognostic biomarker [9]. In our study, copeptin was correlated with hematoma volume, which in turn is associated with clinical severity and outcome. In accordance with this hypothesis, an earlier study showed a correlation between the severity of head injury and copeptin levels on admission[8,26]. In another study, copeptin was associated with lesion size and clinical severity on admission but was still an independent prognostic marker in patients with an acute ischemic stroke [9]. This suggests that copeptin on one hand is associated with the severity of the disease, in ICH patients mirrored by the lesion or the GCS, on the other hand it might still provide additional information related to brain damage.

Copeptin mirrors circulating vasopressin levels and vasopressin itself may also directly influence the clinical course. Data from experimental studies imply that vasopressin plays a role in brain edema formation as blocking of vasopressin receptors attenuates brain edema in ischemic and traumatic mice models[27-29]. The relationship between vasopressin levels and brain edema development has also been demonstrated in a clinical study of head-injured patients[30]. Brain edema formation predicts an unfavorable outcome in ICH[31]. Therefore, copeptin levels might reflect developing or existing brain edema and might therefore be helpful in identifying patients at risk for brain edema formation who could profit from therapeutic interventions, such as the administration of a vasopressin antagonist[28]. A limitation of our study was that we could not monitor brain edema formation and link it with copeptin values, because imaging studies of the brain were not routinely repeated. Hence, the implication of copeptin and brain edema formation in ICH remains hypothetical at the moment.

Copeptin predicted mortality and functional outcome in our ICH cohort and its discriminative power was in the range of GCS, hematoma volume and age, which are known to be strong individual outcome predictors, especially when used in combination[15,16,32]. Other biomarkers have been shown to predict early neurologic deterioration and mortality in ICH patients, i.e. D-dimer [33], glutamate [34], matrix metalloproteinases [3] and protein S100b [35]. Each of these biomarkers reflects a different pathophysiological process which also might have a specific therapeutic implication[5]. In our opinion it is advisable to rely the difficult task of prognostic assessment and treatment decisions upon several parameters. In this context copeptin might have an interesting potential as a new prognostic biomarker in combination with clinical features.

Our study cohort was too small to allow a meaningful multivariate analysis. We thus could not determine whether copeptin is an independent prognostic marker that yields additional information beyond that derivable from other known prognostic factors such as GCS and hematoma volume (both of which are correlated with the copeptin level). These associations were expected since GCS and hematoma volume are strong outcome predictors. It has been shown in a larger cohort in patients with ischemic stroke on the other hand that copeptin is a very strong independent prognostic marker (i.e., independent of age, lesion size, glucose, WBC, CRP and clinical severity on admission) for functional outcome and mortality. Thus it is possible that in a larger cohort copeptin might prove to be an independent marker also in ICH patients [9].

Another limitation of our study is that our cohort included both surgically and non-surgically treated patients. Surgical treatment is unlikely to have influenced the prognosis heavily, however, as the STICH trial did not show any significant benefit of early surgery versus initial conservative treatment[36].

Our study included all patients who presented to the ED within 72 hours after the onset of clinical symptoms, and thus constitutes a heterogeneous population. Due to our limited sample size, we are not able to assess the time effect in our study. In the aforementioned [9] ischemic stroke trial however, a subgroup analysis revealed no difference in the predictive value of the copeptin level depending on whether it was measured 0-3 hours, 3-6 hours, 6-12 hours, 12-24 hours, or 24-72 hours after symptom onset.

## Conclusion

In conclusion, in our cohort of patients with ICH, copeptin was significantly associated with 30-day mortality and with a poor functional outcome at 90 days. If this finding can be validated and confirmed in larger studies, the measurement of copeptin levels may allow together with other clinical parameters improved risk stratification for ICH patients in the future.

## Abbreviations

ICH: Intracerebral Hemorrhage; GCS: Glasgow Coma Scale; AUC: Area Under the Curve; AVP: Arginin-Vasopressin; CRP: C-Reactive Protein; mRS: modified Rankin Scale; BI: Barthel Index.

## Competing interests

NGM is employed by B.R.A.H.M.S., the manufacturer of the copeptinassay (B.R.A.H.M.S. CT-proAVP LIA, B.R.A.H.M.S AG, Hennigsdorf/Berlin, Germany). BM, MCC and PS have served as consultants for B.R.A.H.M.S., from which they have received lecture honoraria, reimbursement of meeting participation fees, and support for research unrelated to the present study. No funding was obtained from commercial sources for this study.

## Authors' contributions

CZ and MK included patients in the study. NGM analyzed the blood samples. PS performed the statistical analysis. MS and AM supported us in collecting the data. BM and MCC participated in the design of the study. All authors read and approved the manuscript.

## Pre-publication history

The pre-publication history for this paper can be accessed here:

http://www.biomedcentral.com/1471-2377/10/34/prepub
